# Surgical treatment of a type IV cystic sacrococcygeal teratoma with intraspinal extension utilizing a posterior-anterior-posterior approach: a case report

**DOI:** 10.1007/s00381-018-3718-9

**Published:** 2018-01-24

**Authors:** Aaron Wessell, David S. Hersh, Cheng-Ying Ho, Kimberly M. Lumpkins, Mari L. A. Groves

**Affiliations:** 1 Department of Neurosurgery, University of Maryland School of Medicine, Baltimore, MD USA; 2 Department of Pathology, University of Maryland School of Medicine, Baltimore, MD USA; 3 Department of Surgery, University of Maryland School of Medicine, Baltimore, MD USA; 4 Division of Pediatric Neurosurgery, Department of Neurosurgery, Johns Hopkins University School of Medicine, Baltimore, MD USA; 5 Johns Hopkins Hospital, 600 N. Wolfe St, Phipps 556, Baltimore, MD 21287 USA

**Keywords:** Sacrococcygeal teratoma, Intraspinal involvement, Sacral spine

## Abstract

Type IV sacrococcygeal teratoma with intraspinal involvement is rare and to our knowledge has not been reported previously in the literature. The authors present the case of a 2-month-old infant with a type IV sacrococcygeal teratoma diagnosed on prenatal ultrasound. Postnatal MRI revealed intraspinal extension through an enlarged sacral neuroforamina on the right side. On surgical exploration, the authors discovered a dorsal cystic tumor involving the sacral spine that extended through an enlarged S4 foramen to a large presacral component. The tumor was successfully removed to achieve a complete en bloc surgical resection. The authors review the epidemiology, pathophysiology, and treatment of sacrococcygeal teratomas with intraspinal extension.

## Introduction

Teratomas are congenital, often benign masses comprised of endoderm, ectoderm, and mesoderm derivatives. While teratomas have been identified in a variety of extra-gonadal and gonadal locations, sacrococcygeal teratomas arising from the coccyx represent the most common subtype [10]. Sacrococcygeal teratomas have an incidence of 1 in 35,000 to 40,000 live births and are typically diagnosed either via prenatal ultrasound, or during a postnatal physical examination [20]. Despite the close anatomic relationship of sacrococcygeal teratomas with the spine, true spinal canal involvement is rare [11, 15, 17, 26, 27, 29, 31]. Here, we report the first case, to our knowledge, of a type IV sacroccoygeal teratoma with intraspinal, extradural extension that underwent an en bloc gross total resection via a posterior-anterior-posterior approach.

## Case report

### Clinical presentation

A female fetus was diagnosed with a pelvic cyst on a prenatal ultrasound that was performed at 37-week gestation. The patient was born at full term via cesarean section following an otherwise uncomplicated pregnancy. During the neonatal period, she tolerated formula despite mild reflux, had regular bowel movements, and was wetting her diaper. Physical examination did not reveal any obvious cutaneous abnormalities, including an exophytic mass or dermal tract.

### Diagnosis

**Fig. 1:**
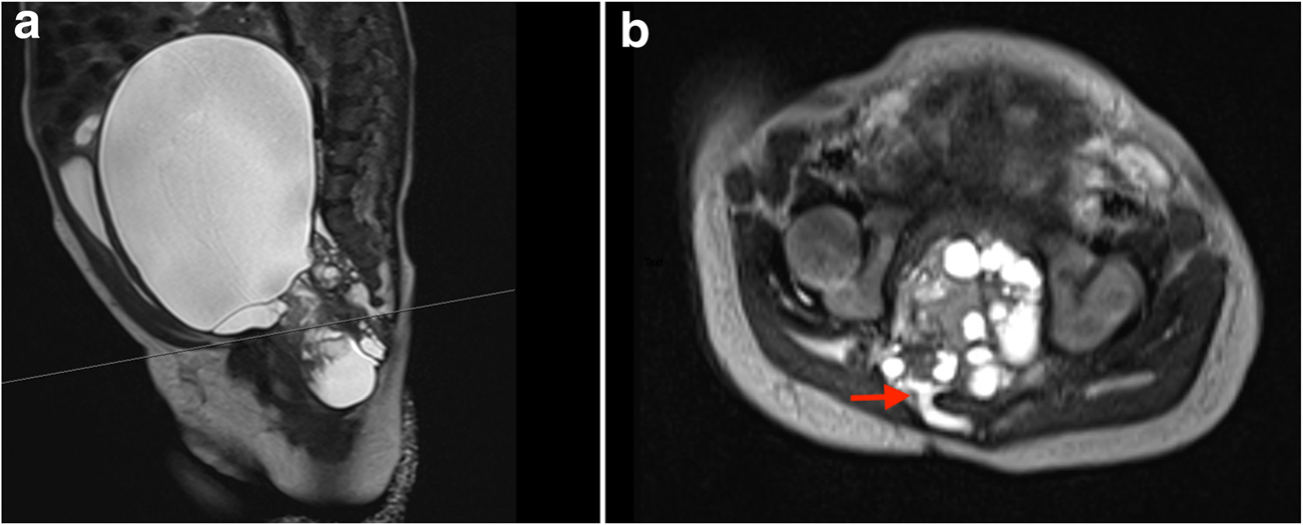
Preoperative, T2-weighted sagittal (left), and axial (right) MR images of the abdomen and pelvis, demonstrating a large cystic mass within the pelvis abutting the sacrum and coccyx. A small posterior extension of the pelvic mass, extending through an enlarged sacral foramen, is observed posterior to the sacral elements (arrow)

Postnatal ultrasound revealed an anechoic, round mass with a thin, hyperechoic wall at the base of the bladder. An MRI of the abdomen, pelvis, and lumbar spine demonstrated a large cystic mass in the pelvis abutting the sacrum and coccyx (Fig. 1). The mass measured 13.2 × 9.7 × 9.6 cm, causing significant mass effect on the adjacent abdominal organs with displacement of the bladder anteriorly. An elongated fluid-filled structure was identified posterior to the sacrum and abutting the coccyx with widening of the right-sided S3 and S4 neuroforamina. Although the lesion appeared to represent a posterior extension of the presacral mass, it was unclear whether the mass was connected to the thecal sac and intradural neural elements. After a multidisciplinary discussion involving a general pediatric surgeon and a pediatric neurosurgeon, the decision was made to proceed with surgical resection and informed consent was obtained.

### Management

The patient was taken to surgery at 2 months of age. She was placed in the prone position and an incision was made from the L5-S1 interspace to the gluteal fold. Partial L5 laminectomies were performed to expose the underlying normal dura. An area of dehiscence was observed caudally, without any evidence of posterior sacral elements. A fibrous layer was transected exposing the tumor, but the area between the cystic tumor and the dura could not be clearly visualized. The tumor was then dissected using bipolary cautery and micro-scissors. A concerted effort was made to maintain the tumor capsule, although the tumor dissection was complicated by the fact that nerve roots were overlying and invested in the tumor capsule bilaterally. The right S4 and S5 nerve roots were sacrificed in an effort to maintain the tumor capsule as these were intimately involved. The tumor was followed to the right S4 foramen where a single pedicle traversed into the presacral component. The pedicle of the tumor capsule was ligated with a 2–0 silk tie, reinforced with small vascular clips, and cut. The intrasacral component of the tumor was removed and a coccygectomy was performed in an effort to preserve bilateral sacral nerve roots as this was thought to be below the extent of the anterior tumor. The thecal sac remained intact, although it was displaced superiorly due to the cystic tumor capsule, and there was no evidence of a cerebrospinal fluid leak.

The posterior wound was closed and the patient was turned to the supine position. A low transverse incision was made across the abdomen and the peritoneal cavity was entered. The bladder had been severely stretched and displaced, and was identified above the level of the umbilicus. The uterus, fallopian tubes, and ovaries were mobilized and the posterior peritoneum was entered in order to uncover the massive retroperitoneal cystic component of the teratoma. The cyst was then freed circumferentially from the overlying peritoneum and approximately 400 ml of clear fluid were drained. Clear tissue planes surrounding the tumor could then be identified, and careful dissection was performed down to the sacral promontory using minimal electrocautery.

Solid components of tumor were identified anterior to the sacral promontory, which appeared to be adherent posteriorly. This solid component of the tumor extended caudally to occupy the entire pelvis up to the sacral promontory. This portion was dissected away from the surrounding tissue but was found to be adherent in the lower right aspect of the sacrum, in the region of the previous intraspinal extension. Bipolar and electrocautery were used to carefully dissect free the surrounding adhesions and feeding vessels, revealing the vascular clips that had been placed on the amputated pedicle of the tumor capsule posteriorly. Adherent tumor remained superior to the prior posterior sacral cut, and in an attempt to completely remove the tumor mass the decision was made to reopen the posterior incision. This was due to limited visualization and concern for the traversing nerve roots. The tissue plane was identified allowing for internal dissection and freeing of the remaining tumor posteriorly. The mass was then successfully removed through the posterior incision. The wound was closed in a multi-layered fashion. The total estimated blood loss during the surgery was approximately 75 ml.

### Outcome

**Fig. 2:**
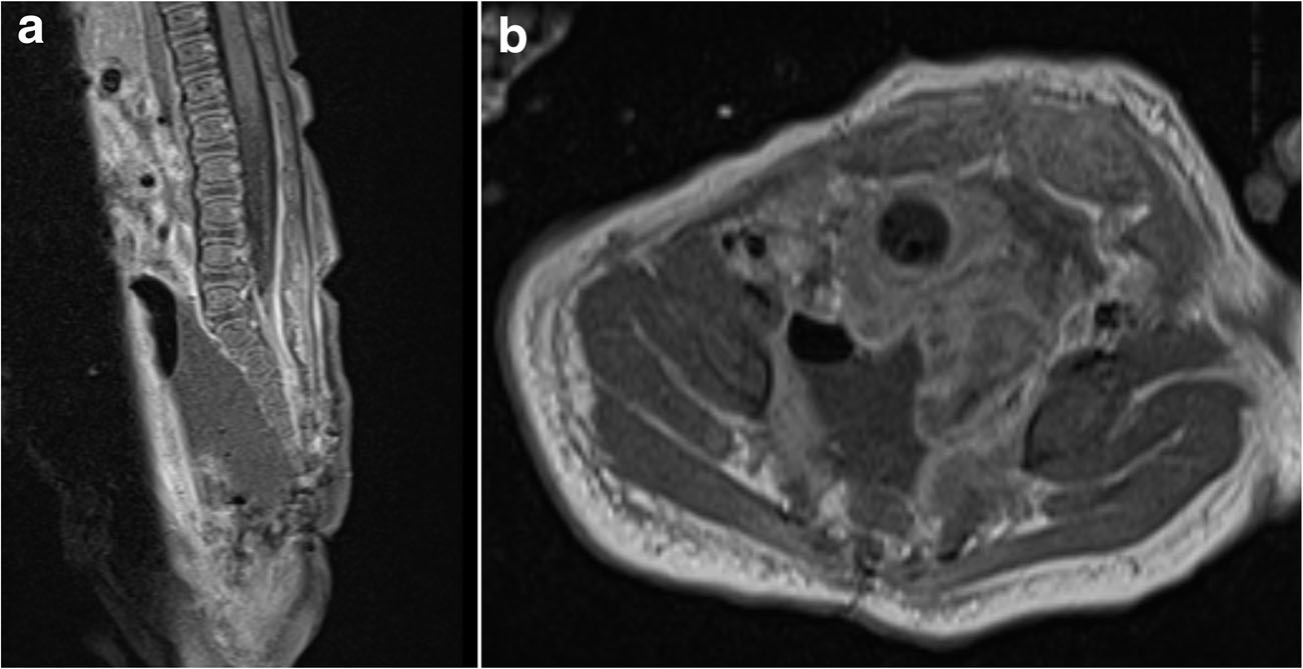
Postoperative, T1-weighted contrast-enhanced sagittal (left), and axial (right) MR images demonstrating a gross total resection without any evidence of residual tumor

**Fig. 3:**
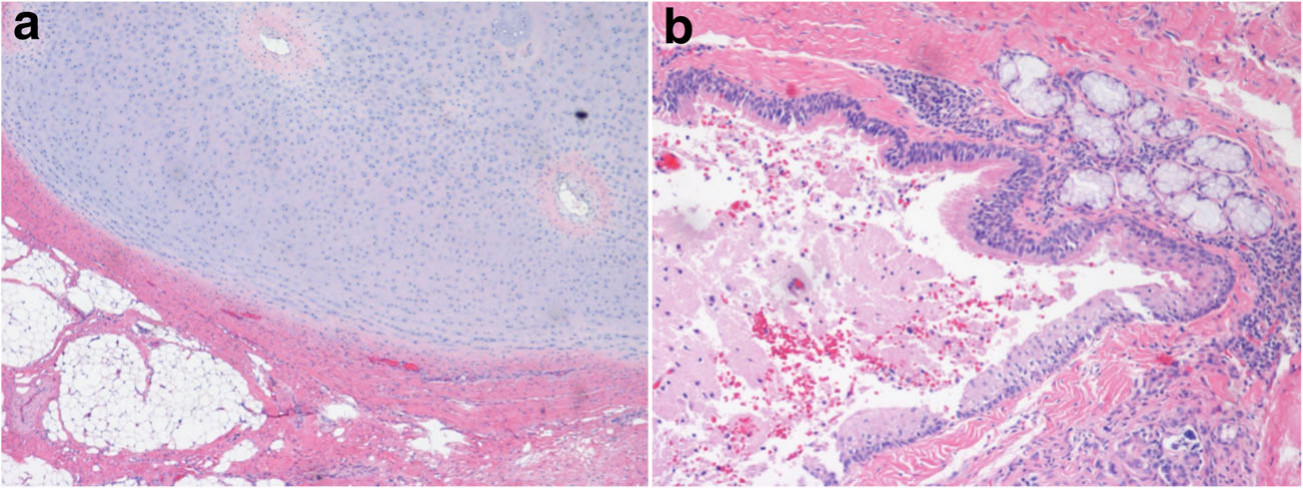
The resected specimen demonstrates hyaline cartilage and fat tissue of mesodermal origin (left), as well as squamous mucosa and respiratory mucosa of endodermal origin (right). H&E, original magnification × 10 (left) and × 20 (right)

Postoperatively, the patient had one self-limited bradycardic episode but had an otherwise uncomplicated course. She underwent an MRI of the lumbar spine with contrast postoperatively, which demonstrated no evidence of residual tumor (Fig. 2). The patient was subsequently discharged home. Final pathology was consistent with a mature cystic sacral teratoma (Fig. 3). At her 5-month follow-up, the patient was doing well. She was feeding, voiding, and stooling normally. The patient was able to stand with assistance and demonstrated good strength in her bilateral lower extremities. A subsequent 10-month follow-up revealed a lower AFP level of 31.4 ng/mL without evidence of recurrent disease on a repeat MRI of the abdomen and pelvis. At 14- and 17-month follow-up appointments, she demonstrated continued progression with the ability to ambulate and pass urine/stool without difficulty.

## Discussion

Sacrococcygeal teratomas represent the most common form of fetal teratoma [17, 29]. The majority of cases present as a benign exophytic mass lesion, identified at birth or on prenatal ultrasound, and can be associated with a variety of other findings, including trisomy 21, club feet, congenital dysplasia/luxation of the hip, hypospadias, congenital urethrovaginal fistula, and obstructive uropathy [6]. Sacrococcygeal teratomas are classified by several criteria including their gross anatomical and histopathological features. The Altman classification categorizes sacrococcygeal teratomas by their varying degrees of internal (intrapelvic) and external components (Table 1) [2]. The Gonzalez-Crussi classification system defines a mature teratoma as grade 0, variations of immature teratomas as grades 1 and 2, and a malignant teratoma as grade 3 [9]. Malignant teratomas can include embryonal carcinomas, yolk sac tumors, germinomas, dysgerminomas, or seminomas [35].

**Table 1:** The Altman classification for sacrococcygeal teratomas

Altman type	Characteristics
I	Sacrococcygeal mass, primarily external
II	Sacroccogyeal mass, primarily external but with significant intrapelvic component
III	Predominantly intrapelvic mass with small external component
IV	Predominantly intrapelvic mass with no external component

The embryologic origin of sacrococcygeal teratomas is not fully understood, although a variety of theories have been proposed. The classic theory attributes tumor formation to aberrant migration of germ cells from the primitive yolk sac [15, 16, 19]. Others suggest that the caudal cell mass, a remnant of the primitive streak and Henson’s node, possesses multipotent mesenchymal progenitor cells, which can give rise to teratomas [13, 15, 16, 28]. Henson’s node resides within the coccyx, which explains the high frequency of tumor formation in this region. Conversely, some authors have proposed the idea that neural crest cells, which are pluriopotent and can differentiate into mesenchymal tissue, are the source of sacroccygeal teratomas and can account for the frequent association with spinal dysraphism [7, 21].

Sacrococcygeal teratomas are typically diagnosed via prenatal ultrasound or upon physical examination shortly after birth. As ultrasound technology has improved, an increasing number of reports have described the prenatal diagnosis of sacrococcygeal teratomas with sonography [22, 34]. Some diagnosed as early as 18-week gestation [4]. One report has even described the diagnosis of a sacrococcygeal terataoma with intraspinal involvement on a prenatal ultrasound [15]. Fetal or newborn MRI can serve as a complement to ultrasound by differentiating between various abdominal, pelvic, and central nervous system lesions. In particular, MRI may facilitate the early diagnosis of type IV sacrococcygeal teratomas [1]. The use of ultrafast fetal MRI has been shown to more accurately characterize intrapelvic and abdominal components of tumors and can readily identify anatomical abnormalities or displacement of intraabdominal or intrapelvic organs [5]. Accurate characterization of tumors enables early treatment during the perinatal period and, in select cases, has prompted prenatal treatment with open fetal resection as early as 21 weeks [5, 25].

The optimal treatment for sacrococcygeal teratomas is en bloc surgical resection, including coccygectomy, within the first 2 months of life [23]. After 2 months, the rate of malignant transformation increases, dramatically complicating subsequent treatment [2, 24]. Teratomas may vary in their consistency and may have cystic and/or solid components, which has technical implications for surgical planning. Lesions comprised of predominantly cystic components are often benign, whereas solid or calcified tumors are more frequently associated with malignancy [2]. Additionally, sacrococcygal teratomas are typically highly vascularized tumors and may exhibit significant arteriovenous shunting, potentially leading to fetal cardiac failure and high mortality [12, 32]. Hemorrhagic complications are the most frequent cause of death among neonates undergoing surgical resection, with a mortality rate in the neonatal period of 16%. The presence of fetal distress, large lesions, and polyhydramnios are all associated with an increased risk of hemorrhage. This can be further exacerbated by a coagulopathy induced by the release of thromboplastins during the trauma of delivery [13]. Poor prognostic features include congestive heart failure, hydrops fetalis and placentomegaly, with an associated mortality of 100% [23].

Nevertheless, patients with sacrococcygeal teratomas can achieve excellent outcomes, particularly if diagnosed early. The 5-year overall survival for sacroccygeal teratomas has been reported at anywhere from 81 to 97%. However, those patients with tumor recurrence have a statistically significant decrease in overall survival compared to those who remain recurrence-free. Several case series have described patients with recurrent lesions [6, 33, 35]. The 5-year overall survival for patients with tumor recurrence ranges from 45 to 58% [3, 8, 14, 30]. Factors found to be associated with recurrence include incomplete resection or spillage of tumor during the primary resection, and immature or malignant histology. Altman grades have not been shown to correlate with tumor recurrence [35].

Functional outcomes, on the other hand, are typically determined by long-term bowel and bladder function. In a review of 47 patients who were treated for sacrococcygeal teratomas as children, 30% were noted to have urinary incontinence as adults and 21% experienced constipation. Risk factors for constipation in adulthood were tumors greater than 10 cm in diameter and Altman type I or type II lesions [18]. While our patient is doing well 17 months postoperatively, she is too young to determine whether she has suffered any long-term complications affecting her bowel or bladder function. She will also require continued follow-up to monitor for any recurrence of her disease.

To our knowledge, there are few documented cases of sacrococcygeal teratomas with intraspinal involvement, and true spinal cord involvement with intradural extension is even less common [11, 15, 17, 26, 27, 29]. The majority of sacrococcygeal teratomas with intraspinal extension are Altman type I or II lesions. Because these lesions are primarily external and located dorsal to the spine, they can often be managed from a sacral, or posterior, surgical approach. We found no prior reported cases of type IV sacrococcygeal teratoma with intraspinal extension. Our case was complicated by a very large (type IV) primarily presacral lesion with dorsal intraspinal extension, necessitating a combined surgical approach in order to provide optimal visualization of the tumor while avoiding undue traction on the nerve roots and the risk of neurologic injury while working ventrally.

In this case, specifically, the collaboration between neurosurgery and pediatric surgery colleagues was essential, as a combined surgical approach provided optimal visualization of the tumor, minimization of blood loss, and allowed for gross total resection while avoiding neurologic or genitourinary injury. A comprehensive preoperative evaluation, including MRI, is required in order understand the extent of one’s lesion and properly plan for what can potentially be a high-risk surgical operation.

## Conclusions

Sacrococcygeal teratomas with intraspinal extension are rare. Complex lesions with a combination of intrapelvic and external components may require the incorporation of hybrid surgical approaches and interdisciplinary collaboration in order to achieve an adequate resection. We successfully managed a type IV sacrococcygeal teratoma with intraspinal extension using a combined posterior-anterior-posterior approach resulting in complete oncologic resection and limited neurologic morbidity.

## References

[1] Adekola H, Mody S, Bronshtein E, Puder K, Abramowicz JS (2015). The clinical relevance of fetal MRI in the diagnosis of a type IV cystic sacrococcygeal teratoma—a review. Fetal Pediatr Pathol.

[2] Altman RP, Randolph JG, Lilly JR (1974). Sacrococcygeal teratoma: American Academy of Pediatrics Survey. J Pediatr Surg.

[3] Bilik R, Shandling B, Pope M, Thorner P, Weitzman S, Ein SH (1993). Malignant bening neonatal sacrococcygeal teratoma. J Pediatr Surg.

[4] Burgess I, Hines B, Stevenson P (1998). Cystic type IV sacrococcygeal teratoma detected at 18-week prenatal ultrasound. Ultrasound Obstet Gynecol.

[5] Danzer E, Hubbard AM, Hedrick HL, Johnson MP, Wilson RD, Howell LD, Flake AW, Adzick NS (2006). Diagnosis and characterization of fetal sacrococcygeal teratoma with prenatal MRI. AJR Am J Roentgenol.

[6] De Backer A, Madern GC, Hakvoort-Cammel HP, Oosterhuis JW, Hazebroek FW (2006). Study of the factors associated with recurrence in children with sacrococcygeal teratoma. J Pediatr Surg.

[7] Dupin E, Sommer L (2012). Neural crest progenitors and stem cells: from early development to adulthood. Dev Biol.

[8] Ein SH, Adeyemi S, Mancer K (1980). Benign sacrococcygeal teratomas in infants and children: a 25 year review. Ann Surg.

[9] Gonzalez-Crussi F, Winkler RF, Mirkin DL (1978). Sacrococcygeal teratomas in infants and children: relationship of histology and prognosis in 40 cases. Arch Pathol Lab Med.

[10] Gross RE, Clatworthy HW, Irving IA (1951). Sacrococcygeal teratomas in infants and children. Surg Obstet Gynecol.

[11] Guvenc BH, Etus V, Muezzinoglu B (2006). Lumbar teratoma presenting intradural and extramedullary extension in a neonate. Spine J.

[12] Hecker K, Hackelöer BJ (1996). Intrauterine endoscopic laser surgery for fetal sacroccygeal teratoma. Lancet.

[13] Herman TE, Siegel MJ (2002). Cystic type IV sacrococcygeal teratoma. J Perinatol.

[14] Huddart S, Mann JR, Robinson RF, Imeson J, Gornall P, Sokal M, Gray E, McKeever P, Oakhill A, Children's Cancer Study Group (2003). Sacrococcygeal teratomas: the UK Children’s Cancer Study Group’s experience. Pediatr Surg Int.

[15] Jelin E, Jelin C, Lee H (2009). Sacrococcygeal teratoma with spinal canal invasion prenatally diagnosed. J Pediatr Surg.

[16] Kalani MY, Iyer S, Coons SW, Smith KA (2012). Spinal intradural teratomas: developmental programs gone awry?. Neurosurg Focus.

[17] Koen JL, McLendon RE, George TM (1998). Intradural spinal teratoma: evidence for a dysembryogenic origin. Report of four cases. J Neurosurg.

[18] Kremer ME, Derikx JP, van Baren R, Heij HA, Wijnen MH, Wijnen RM, van der Zee DC, van Heurn EL (2016). Patient-related defecation and micturition problems among adults treated for sacrococcygeal teratoma during childhood—the need for new surveillance strategies. Pediatr Blood Cancer.

[19] Lu YH, Wang HH, Lirng JF, Guo WY, Wong TT, Teng MM, Chang FC, Chang CY (2013). Unusual giant intraspinal teratoma in an infant. J Chin Med Assoc.

[20] Moore SW, Satgé D, Sasco AJ, Zimmerman A, Plaschkes J (2003). The epidemiology of neonatal tumours. Report of an international working group. Pediatr Surg Int.

[21] Morikawa S, Mabuchi Y, Niibe K, Suzuki S, Nagoshi N, Sunabori T, Shimmura S, Nagai Y, Nakagawa T, Okano H, Matsuzaki Y (2009). Development of mesenchymal stem cells partially originate from the neural crest. Biochem Biophys Res Commun.

[22] Morrow RJ, Whittle M, McNay MB, Cameron AD, Raine PA, Gibson AA (1990). Prenatal diagnosis of an intra-abdominal sacrococcygeal teratoma. Prenat Diagn.

[23] Murphy JJ, Blair GK, Fraser GC (1992). Coagulopathy associated with large sacrococcygeal teratomas. J Pediatr Surg.

[24] Noseworthy J, Lack EE, Kozakewich HP, Vawter GF, Welch KJ (1981). Sacrococcygeal germ cell tumors in childhood: an updated experience with 118 patients. J Pediatr Surg.

[25] Perrone EE, Jarboe MD, Maher CO, Berman DR, Ladino-Torres M, Kreutzman J, Treadwell MC, Mychaliska GB (2017) Early delivery of sacrococcygeal teratoma with intraspinal extension. Fetal Diagn Ther 10.1159/000472714 28463844

[26] Powell RW, Webber ED, Manci EA (1993). Intradural extension of a sacrococcygeal teratoma. J Pediatr Surg.

[27] Ribeiro PR, Guys JM, Lena G (1999). Sacrococcygeal teratoma with an intradural and extramedullary extension in a neonate: case report. Neurosurgery.

[28] Schey WL, Shkolnik A, White H (1977). Clinical and radiographic considerations of sacrococcygeal teratomas: an analysis of 26 new cases and review of the literature. Radiology.

[29] Shahjouei S, Hanaei S, Nejat F, Monajemzadeh M, Khashab ME (2015). Sacrococcygeal teratoma with intradural extension: case report. J Neurosurg Pediatr.

[30] Shanbhogue L, Bianchi A, Doig CM, Gough CS (1990). Management of benign sacrococcygeal teratoma: reducing mortality and morbidity. Pediatr Surg Int.

[31] Teal LN, Angtuaco T, Jimenez JF, Quirk JG (1988). Fetal teratomas: antenatal diagnosis and clinical management. J Clin Ultrasound.

[32] Veschambre P, Wartanian R, Lebouvier B, Rosenau L, Lepinard C, Denis A (1993). Antenatal prognostic factors in sacrococcygeal teratomas [in French]. Rev Fr Gynécol Obstét.

[33] Wakhlu A, Misra S, Tandon RK, Wakhlu AK (2002). Sacrococcygeal teratoma. Pediatr Surg Int.

[34] Winderl LM, Silverman RK (1997). Prenatal identification of a completely cystic internal sacrococcygeal teratoma (type IV). Ultrasound Obstet Gynecol.

[35] Yao W, Li K, Zheng S, Dong K, Xiao X (2014). Analysis of recurrence risks for sacrococcygeal teratoma in children. J Pediatr Surg.

